# The case for using mapped exonic non-duplicate reads when reporting RNA-sequencing depth: examples from pediatric cancer datasets

**DOI:** 10.1093/gigascience/giab011

**Published:** 2021-03-13

**Authors:** Holly C Beale, Jacquelyn M Roger, Matthew A Cattle, Liam T McKay, Drew K A Thompson, Katrina Learned, A Geoffrey Lyle, Ellen T Kephart, Rob Currie, Du Linh Lam, Lauren Sanders, Jacob Pfeil, John Vivian, Isabel Bjork, Sofie R Salama, David Haussler, Olena M Vaske

**Affiliations:** UC Santa Cruz, Molecular, Cell and Developmental Biology, 1156 High Street, Santa Cruz, CA 95064, USA; UC Santa Cruz, Genomics Institute, 1156 High Street, Santa Cruz, CA 95064, USA; UC Santa Cruz, School of Engineering, 1156 High Street, Santa Cruz, CA 95064, USA; UC Santa Cruz, School of Engineering, 1156 High Street, Santa Cruz, CA 95064, USA; UC Santa Cruz, School of Engineering, 1156 High Street, Santa Cruz, CA 95064, USA; UC Santa Cruz, School of Engineering, 1156 High Street, Santa Cruz, CA 95064, USA; UC Santa Cruz, Genomics Institute, 1156 High Street, Santa Cruz, CA 95064, USA; UC Santa Cruz, Molecular, Cell and Developmental Biology, 1156 High Street, Santa Cruz, CA 95064, USA; UC Santa Cruz, Genomics Institute, 1156 High Street, Santa Cruz, CA 95064, USA; UC Santa Cruz, Genomics Institute, 1156 High Street, Santa Cruz, CA 95064, USA; UC Santa Cruz, Genomics Institute, 1156 High Street, Santa Cruz, CA 95064, USA; UC Santa Cruz, Genomics Institute, 1156 High Street, Santa Cruz, CA 95064, USA; UC Santa Cruz, Molecular, Cell and Developmental Biology, 1156 High Street, Santa Cruz, CA 95064, USA; UC Santa Cruz, Genomics Institute, 1156 High Street, Santa Cruz, CA 95064, USA; UC Santa Cruz, Genomics Institute, 1156 High Street, Santa Cruz, CA 95064, USA; UC Santa Cruz, Genomics Institute, 1156 High Street, Santa Cruz, CA 95064, USA; UC Santa Cruz, Department of Biomolecular Engineering, 1156 High Street, Santa Cruz, CA 95064, USA; Howard Hughes Medical Institute, 1156 High Street, Santa Cruz, CA 95064, USA; UC Santa Cruz, Department of Biomolecular Engineering, 1156 High Street, Santa Cruz, CA 95064, USA; Howard Hughes Medical Institute, 1156 High Street, Santa Cruz, CA 95064, USA; UC Santa Cruz, Molecular, Cell and Developmental Biology, 1156 High Street, Santa Cruz, CA 95064, USA; UC Santa Cruz, Genomics Institute, 1156 High Street, Santa Cruz, CA 95064, USA

**Keywords:** RNA-Seq, sequencing, depth, duplicate, unmapped, exonic, quality

## Abstract

**Background:**

The reproducibility of gene expression measured by RNA sequencing (RNA-Seq) is dependent on the sequencing depth. While unmapped or non-exonic reads do not contribute to gene expression quantification, duplicate reads contribute to the quantification but are not informative for reproducibility. We show that mapped, exonic, non-duplicate (MEND) reads are a useful measure of reproducibility of RNA-Seq datasets used for gene expression analysis.

**Findings:**

In bulk RNA-Seq datasets from 2,179 tumors in 48 cohorts, the fraction of reads that contribute to the reproducibility of gene expression analysis varies greatly. Unmapped reads constitute 1–77% of all reads (median [IQR], 3% [3–6%]); duplicate reads constitute 3–100% of mapped reads (median [IQR], 27% [13–43%]); and non-exonic reads constitute 4–97% of mapped, non-duplicate reads (median [IQR], 25% [16–37%]). MEND reads constitute 0–79% of total reads (median [IQR], 50% [30–61%]).

**Conclusions:**

Because not all reads in an RNA-Seq dataset are informative for reproducibility of gene expression measurements and the fraction of reads that are informative varies, we propose reporting a dataset's sequencing depth in MEND reads, which definitively inform the reproducibility of gene expression, rather than total, mapped, or exonic reads. We provide a Docker image containing (i) the existing required tools (RSeQC, sambamba, and samblaster) and (ii) a custom script to calculate MEND reads from RNA-Seq data files. We recommend that all RNA-Seq gene expression experiments, sensitivity studies, and depth recommendations use MEND units for sequencing depth.

## Background

Assessing the reproducibility of RNA sequencing (RNA-Seq) gene expression measurements has been a priority since the development of the assay [1,2]. The amount of sequencing generated from all regions of the genome for a dataset generated from 1 biological sample is called the depth of sequence for that dataset. Seminal studies showed the following 3 effects of increasing the depth of sequencing: the convergence of measurements of the expression of individual genes in a single dataset to a consistent value [2], the increase in the number of true-positive differentially expressed genes in cross-dataset comparisons [1], and an increase in correlations across platforms of fold-change measurements in cross-dataset comparisons [1]. These show how reproducibility within a dataset, between datasets, and across platforms all depend on the depth of sequence. The degree of reproducibility required depends on the experimental design; finding large fold changes across genes requires less reproducibility in gene expression values than finding smaller differences between isoforms. For comparing gene expression measurements between datasets, ENCODE recommends a minimum of 30 million mapped reads [3]; the Genetic European Variation in Disease (GEUVADIS) consortium study had a minimum goal of 20 million reads [4].

However, RNA-Seq data are not homogenous. Of the tens of millions of sequences (reads) in a typical RNA-Seq dataset, some reads cannot be mapped back to the reference transcriptome. Others map to genome regions outside of exons or have been duplicated by PCR during the library construction process or sequencing. Nearly all methods for quantifying gene expression in bulk RNA-Seq data count reads that align to exons in a gene; thus, unmapped and non-exonic reads do not contribute to measurements and are consequently uninformative [5,6]. Therefore, if the fraction of uninformative reads varies between datasets, using the total number of reads as a proxy for RNA-Seq gene expression reproducibility can result in inflated reproducibility estimates.

Duplicate reads may be due to either highly abundant transcripts or technical artifacts. The process of preparing RNA-Seq libraries involves PCR amplification. This step can generate duplicated identical or nearly identical reads, especially if the input amount is low. While the original read represents gene expression in the experimental system, the artifactual duplicate reads do not. However, duplicate reads are also generated by very highly expressed genes because each gene has a finite number of unique read sequences that can be generated from it [7]. Previous studies have shown that many duplicates in high-quality datasets reflect gene expression, and there is strong evidence that duplicates should not be removed for the purpose of measuring the expression of individual genes [8,9]. Below we investigate the value of excluding duplicates at the dataset level when reporting on the dataset's sequencing depth.

Here we analyze 2,179 bulk, paired-end, polyA-selected RNA-Seq datasets to characterize the read types present in the datasets and evaluate what fraction of each dataset is unequivocally relevant to the reproducibility of gene expression measurements.

## Methods

### MEND read counting method

Quantification of mapped, exonic, non-duplicate (MEND) reads was previously described [10]. Briefly, input to the program that computes MEND is a genome-aligned bam file containing RNA-Seq read data. Duplicates are marked with Samblaster v0.1.22 (Samblaster, RRID:SCR_000468) [11], and the RSeQC v2.7.10 [12] script read_distribution.py quantifies exonic read and tag counts, excluding quality control (QC) fail and duplicate reads, as well as secondary alignments. The script parseReadDist.R, which we wrote, estimates the number of MEND reads based on RSeQC output by summing the tag counts in CDS exons, 5′ untranslated region (UTR) exons, and 3′ UTR exons and multiplying by reads per tag. Because a pair of reads provides information about 2 nearby sequences, read counts are reported in pairs. For example, 20 million reads means that there are 20 million pairs of reads. The process for estimating MEND read counts is available as a stand-alone Docker image [13] and can be executed on CodeOcean [14]. The source code is freely available on GitHub [15].

### Data description

Here we discuss 2,179 publicly available, polyA-selected, bulk RNA-Seq datasets that we gathered for the RNA-Seq compendium [16] used for comparative single-patient analysis [10]. Accession numbers, clinical metadata, and read counts for each dataset are in Supplementary Table S1. Repository and cohort information is aggregated in Supplementary Tables S2 and S3.

Of the 2,179 datasets, 2,018 were generated from pediatric/adolescent/young adult cancer tumors, 66 were from adult cancer tumors, and 95 were from cancer tumors of individuals with unknown ages, where adults are defined as being >30 years of age. Of the 1,692 datasets with reported sex of the patient, 42% were female and 58% were male. Of the 602 datasets with reported race of the patient, 27 were Asian, 70 were Black/African American, 3 were Native Hawaiian or Other Pacific Islander, 494 were white, 1 identified as “non-white" and 7 as “other" without further specification. None were American Indian or Alaskan Native. Of 861 datasets with reported results of the patient's Hispanic or Latino identity, 128 were Hispanic or Latino. The source tumors represent a variety of hematologic and solid malignant neoplasms (Table 1).

**Table 1.** Diseases represented in studied datasets

Disease	No. (%)
Acute lymphoblastic leukemia	680 (31.2)
Acute myeloid leukemia	221 (10.1)
Medulloblastoma	201 (9.2)
Glioma	193 (8.9)
Osteosarcoma	157 (7.2)
Acute megakaryoblastic leukemia	103 (4.7)
Ependymoma	98 (4.5)
Ewing sarcoma	70 (3.2)
Rhabdoid tumor	65 (3.0)
Rhabdomyosarcoma	53 (2.4)
Lymphoma	49 (2.2)
Embryonal rhabdomyosarcoma	42 (1.9)
Alveolar rhabdomyosarcoma	40 (1.8)
Glioblastoma multiforme	29 (1.3)
Choroid plexus carcinoma	25 (1.1)
Synovial sarcoma	22 (1.0)
Other	131 (6.0)

**Figure 1.**
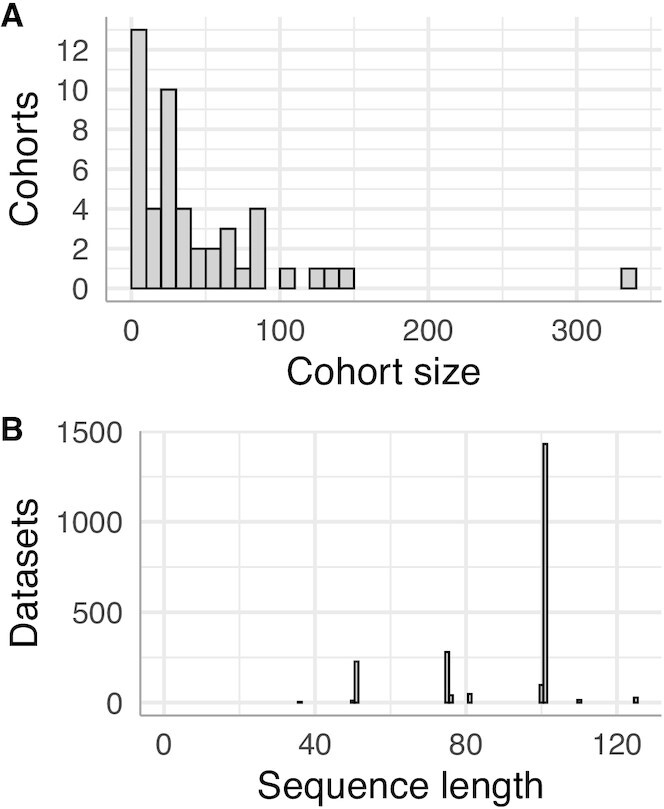
RNA-Seq datasets from 48 tumor cohorts with a variety of read lengths were analyzed. A. Distribution of number of datasets per cohort. B. Distribution of length of paired-end reads in this study.

The datasets came from 5 repositories (Supplementary Table S2). Each was assigned to a cohort based on (i) project accession (for EGA and SRA datasets), (ii) disease substudy for National Cancer Institute Therapeutically Applicable Research to Generate Effective Treatments (TARGET), or (iii) disease for datasets in the St Jude Cloud. Cohorts were assigned IDs in descending order of size. Cohort assignments were intended to approximate a typical sequencing project performed by 1 research group at 1 sequencing center. The cohorts range in size from 3 to 337 datasets (Fig. 1A); the median number of datasets in a cohort is 24.5.

All libraries were prepared with polyA selection. All data were generated via paired-end Illumina sequencing technology. The reported sequencing methods for 1,133 datasets included the model of Illumina sequencer used. The models included the Genome Analyzer (II, IIx, and unspecified); the HiSeq (2000, 2500, and unspecified); and the Next Seq 500. The median sequence length is 101 bases (Fig. 1B).

### Data analysis

RNA-Seq read data were aligned to the genome with the TOIL RNA-Seq pipeline previously described [17]. Briefly, adapters were removed with CutAdapt v1.9 (CutAdapt, RRID:SCR_011841) [18]. Reads were then aligned with STAR v2.4.2a (STAR, RRID:SCR_015899) [19] with indices based on GRCh38 and gencode v23. RSEM v1.2.25 was used to quantify gene expression. The source code of the pipeline is available [20]. MEND read counts were calculated with MEND qc release v1.1.1.

Read count and gene expression analysis was conducted with the R programming language, using the following packages: tidyverse, janitor, knitr, corrr, cowplot, RColorBrewer, pander, kableExtra, and snakecase [21–30]. The source code used to generate the figures and statements in this article is available on Github [31] and can be run and modified on Code Ocean [32].

## Results

### Read types in RNA-Seq data

**Figure 2.**
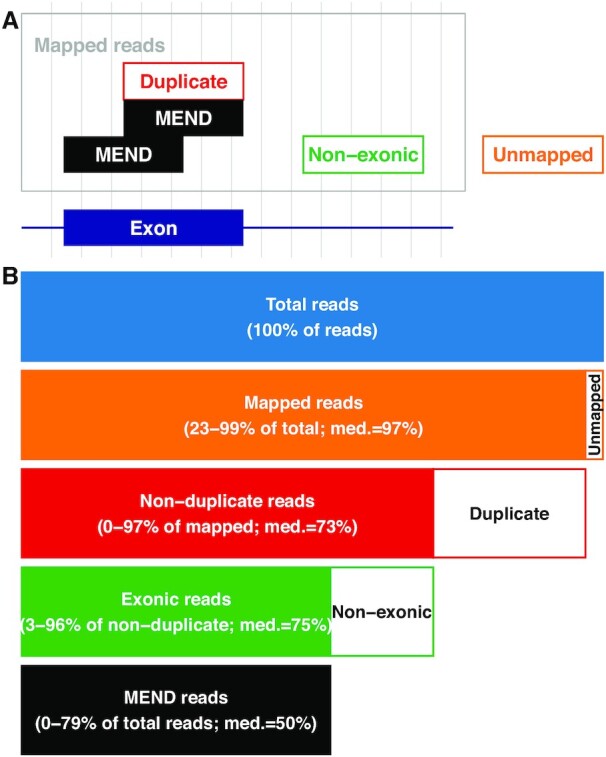
RNA-Seq datasets include 4 main types of sequencing reads. A. Simplified schematic illustrating read types. The X axis (blue) is a genomic locus containing an exon. The other boxes each represent 1 sequencing read. Two of 5 reads are MEND reads. Other reads do not map to the genome (unmapped; orange border), map to a non-exonic region of the genome (non-exonic; green border), or are duplicates of other reads (duplicate; red border). The MEND reads (black) fit none of these categories and are most informative for determining the reproducibility of gene expression quantification. B. Schematic illustrating read type quantification. Bars representing uninformative reads are white with a colored border. For each informative fraction, the range and median (med.) are reported.

We interrogated the read types present in our RNA-Seq datasets as defined by our gene expression quantification pipeline (Fig. 2A). We obtained the number of total and mapped reads from the aligner log. We marked duplicates in the aligned BAM file, and counted them, along with exonic reads, using RSeQC. Duplicate reads are reported as a fraction of mapped reads, and exonic reads are reported as a fraction of non-duplicate reads. The datasets ranged in total sequence depth from 0.2 to 668 million reads, with a median value of 61 million and an interquartile range (IQR) of 49–102 million.

**Figure 3.**
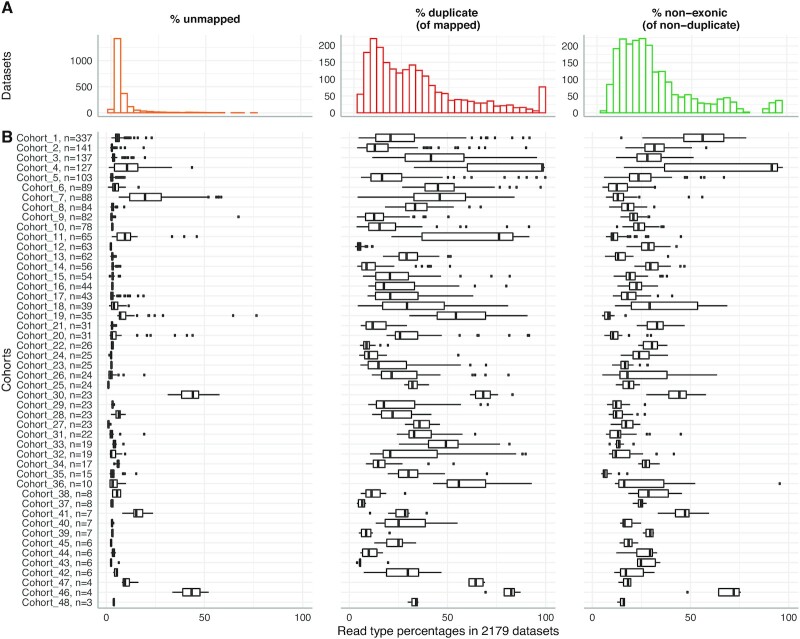
Read type fractions vary within and between cohorts. A. The percent distribution of different uninformative read types observed in 2,179 datasets. B. The percentage of read types observed in cohorts, annotated with the number of datasets in the cohort.

Most RNA-Seq datasets contain a small percentage of unmapped reads (Fig. 2B). While the fraction of unmapped reads in the 2,179 datasets ranges from 1 to 77%, the median value is 3% and the IQR is 3–6%. The distribution is left-skewed with a long right tail (Fig. 3A). In 77 datasets, >25% of reads are unmapped. The value of excluding unmapped reads from sequencing depth read counts is self-evident because these reads do not correspond to any known expressed gene and do not contribute to gene expression measurements. Including those reads in any measure of the reproducibility of gene expression measurement would misguide the researcher.

The percentage of mapped reads that are duplicate reads (“percent duplicates'') is more varied than the percentage of unmapped reads. Duplicate reads constitute 3–100% of mapped reads (median [IQR], 27% [13–43%]). 426 datasets have >50% duplicates (Fig. 3A). The duplicate read fraction varies within and between cohorts (Fig. 3B). For example, Cohort 4 is characterized by high duplicate fractions, with 72 of the 127 datasets having >98% duplicates. Remarkably, these 72 datasets all identify <100 expressed genes. However, Cohort 4 does not account for all datasets with high duplicate fractions: 20 datasets in other cohorts have >90% duplicates. Even cohorts with generally low duplicate fractions can contain anomalous datasets; of the 41 cohorts with a median of <50% duplicates, 26 contain ≥1 dataset with >50% duplicates.

**Figure 4.**
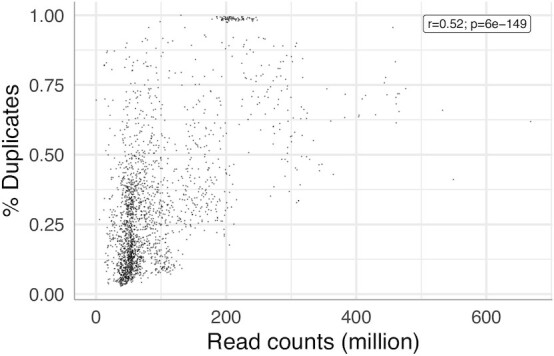
Duplicate fraction is not completely explained by total read depth. The Spearman correlation and *P*-value are shown. Many datasets have very different duplicate fractions in spite of similar total read counts; n = 2,179.

If duplicate reads were only a function of datasets being especially deeply sequenced, we would expect datasets with deeper sequencing to have a greater fraction of duplicate reads than all datasets with lower depth of sequence.The total sequencing depth has a 0.52 Spearman correlation with the fraction of duplicate reads (Fig. 4). The incomplete explanation of duplicate fractions by sequence depth is consistent with Fu et al. [8] and with the large number of datasets in Fig. 4 that have very different duplicate fractions in spite of similar total read counts. The fraction of duplicate reads cannot be inferred from the total read depth.

Like percent of duplicates, the percent of non-exonic reads among all mapped, non-duplicate reads (“percent non-exonic”) has a broad distribution compared to other read type fractions, ranging from 4% to 97% with a median [IQR] of 25% [16–37%]. There are 330 datasets that have a fraction of non-exonic reads >50%. Therefore, the percentage of duplicate reads or non-exonic reads among all mapped, non-duplicate reads is not directly informative about the dataset's gene expression measurements.

### Computing requirements for MEND pipeline

We recorded the time required to run our most recent pipelines on computers with 64 GB of memory and 12 VCPU. The 382 RNA-Seq datasets examined were obtained from SRA and EGA and had reads that were 100 bases in length. For datasets within 10 million total reads of the median total read size in our survey of 2,179 datasets (61 million total reads), the median duration is 290 minutes (4.8 hours) for the expression pipeline and 143 minutes (2.4 hours) for the MEND pipeline.

## Conclusion

Researchers wish to know that their data are sufficient for making reproducible measurements. For RNA-Seq experiments, they often wish to know whether the dataset is sufficient for reproducibly measuring expression of known genes. Here we show that the fraction of relevant content of an RNA-Seq dataset (percent of MEND reads) varies substantially within and between cohorts, and thus should be measured in each dataset.

This work was performed using data from pediatric tumor datasets as part of the development of our comparative RNA-Seq assay for patients with pediatric cancer [10,16]. Because the factors that reduce the quality of RNA-Seq datasets (e.g., degradation, low input amounts, contamination, and low base quality) are not specific to pediatric cancer datasets, we predict that other kinds of RNA-Seq datasets would also show compositional variability. The MEND read counting tool is independent of species and genome version; it can be used on any bulk RNA-Seq dataset.

Previous studies have shown that paired-end libraries contain relatively few artifactual duplicate reads [7,9]. However, these studies are typically conducted on high-quality datasets from a single source. For example, Parekh et al. [9] base their conclusions on analysis of paired-end datasets with a range of duplicates of 6–19%. In our survey of real-world data, Cohort 4 contains 72 datasets with >98% duplicates, and 20 more datasets from other cohorts contain >90% duplicates.

There are several reasons why a survey of this breadth has not been previously performed. Obtaining and processing clinical datasets from multiple sources is an intensive effort [33]. Access to tumor datasets is usually controlled, and obtaining the 48 cohorts that we report on here required multiple legal agreements [33]. Analyzing read types requires genome-aligned reads; the files containing genome-aligned reads are large and are not generated when using the much faster pipelines that quantify gene expression via pseudoalignment. Large RNA-Seq cohorts such as Genotype-Tissue Expression (GTEx) and The Cancer Genome Atlas use consistent methods and exclude datasets that fail their stringent and consistent QC [34,35]. They lack the kind of heterogeneity observed in data cohorts gathered from diverse sources. In short, generating these data for >2,000 datasets is time-consuming and expensive and requires staff with diverse expertise.

Measuring the number of MEND reads in a dataset is specific to the alignment parameters and gene model. We use Gencode v23, which is inclusive, defining >60,000 genes. By default, the aligner we use, STAR, defines reads that map to as many as 20 positions as mappable. If we changed our pipeline, asking STAR to exclude reads mapping to >2 positions and using a more conservative gene model with 30,000 genes, the same dataset would have fewer MEND reads owing to the loss of reads that map to too many places or map only to regions newly defined as non-exonic.

In addition to being sensitive to reference files, MEND counts are slow to compute, increasing the duration of our RNA-Seq pipeline by 50%. It would be valuable to create a faster utility that takes raw reads rather than aligned reads as input. The reference-dependence could also be addressed by including a default set of references, with support for alternate ones.

Researchers planning RNA-Seq experiments look for guidance on how much sequencing their experiment requires. For comparing gene expression measurements between datasets, ENCODE recommends a minimum of 30 million mapped reads [3]; the GEUVADIS consortium study had a minimum goal of 20 million reads [4]. However, of the 2,078 datasets in this study with >30 million mapped reads, 16% contain <25% informative (MEND) reads. We speculate that these guidelines were not intended to include those datasets, some of which measure <100 genes. Because the median fraction of MEND reads in our survey was 50%, we recommend that a user who, e.g., wants to follow the ENCODE recommendation of a depth of 30 million mapped reads ensure that they have ≥15.5 million MEND reads (a dataset with 30 million mapped reads typically has 1 million additional unmapped reads). A total of 13% (261) of the datasets in our study that satisfy the ENCODE guideline have <50% MEND reads; 6% (134) have <10% MEND reads.

On the basis of these results, we recommend that (i) publications reporting the results of an RNA-Seq study with gene expression applications should report the depth of sequence as the number of MEND reads present in each dataset; (ii) sensitivity studies should include read type fractions and report on the relationship between MEND reads and the measured outcome; and (iii) sequencing depth recommendations should be based on MEND reads rather than total or total mapped reads.

## Availability of Supporting Source Code and Requirements

Project name: MEND QC

Project home page: https://github.com/UCSC-Treehouse/mend_qc

Operating system(s): Platform independent

Programming language: Bash and R

Other requirements: Docker

License: MIT

RRID:SCR_020934

## Data Availability

Accession numbers, clinical data, and read counts for 2,179 publicly available, bulk RNA-Seq datasets are in Supplementary Table S1. The sequence data are controlled access and can be requested via the accession numbers at the repositories in Supplementary Table S2. Code snapshots and tabular data are available from the *GigaScience* GigaDB repository [36].

## Additional Files

Supplementary Table S1. Accession numbers, clinical data, and read counts for 2,179 publicly available, bulk RNA-Seq datasets. The accession numbers are the definitive sources; the DOI links to citations are provided for convenience.

Supplementary Table S2. Sequence data repositories, URLs, and abbreviations.

Supplementary Table S3. Cohort names, code, repositories, and dataset counts.

## Abbreviations

CDS: coding sequence; DOI: digital object identifier; EGA: European Genome-Phenome Archive; GEUVADIS: Genetic European Variation in Disease; GTEx: Genotype-Tissue Expression; IQR: interquartile range; med.: median; MEND: Mapped, Exonic, Non-Duplicate; QC: quality control; RNA-Seq: RNA sequencing; SRA: Sequence Read Archive; UTR: untranslated region; VCPU: virtual central processing unit.

## Ethics

The UCSC Institutional Review Board (IRB) has determined that our use of previously released sequence data does not constitute human subject research, and therefore does not require an IRB review.

## Competing Interests

The authors declare that they have no competing interests.

## Funding

This study was funded by American Association for Cancer Research NextGen Grant for Transformative Cancer Research Award (O.M.V.), Emily Beazley Kures for Kids Fund St. Baldrick’s Consortium Grant, Alex's Lemonade Stand Foundation for Childhood Cancer Research, Unravel Pediatric Cancer, Team G Childhood Cancer Foundation, California Initiative to Advance Precision Medicine, Live for Others Foundation, The Schmidt Futures Foundation (D.H.). D.H. is a Howard Hughes Medical Institute Investigator. O.M.V. holds the Colligan Presidential Chair in Pediatric Genomics.

## Authors' Contributions

Analysis and manuscript authorship: H.C.B., J.M.R., M.A.C., L.T.M., D.K.A.T.

MEND pipeline development and integration and manuscript review: R.C., D.L.L., J.V.

Data access, data processing, and manuscript review: K.L., E.T.K., L.S., J.P., A.G.L., and I.B.

Funding, scientific oversight, and manuscript review: D.H., S.R.S., and O.M.V.

## Supplementary Material

giab011_GIGA-D-20-00263_Original_Submission

giab011_GIGA-D-20-00263_Revision_1

giab011_GIGA-D-20-00263_Revision_2

giab011_Response_to_Reviewer_Comments_Original_Submission

giab011_Response_to_Reviewer_Comments_Revision_1

giab011_Reviewer_1_Report_Original_Submission Alexandra Sexton Oates -- 9/27/2020 Reviewed

giab011_Reviewer_2_Report_Original_Submission Mark Ziemann, PhD -- 9/28/2020 Reviewed

giab011_Reviewer_2_Report_Revision_1 Mark Ziemann, PhD -- 1/6/2021 Reviewed

giab011_Supplemental_Tables
