# Supplementary material for: The case for using mapped exonic non-duplicate reads when reporting RNA-sequencing depth: examples from pediatric cancer datasets
Source: Gigascience. 2021 Mar 13;10(3):giab011. doi: 10.1093/gigascience/giab011 (PMC7955155; doi:10.1093/gigascience/giab011)
Supplement: giab011_GIGA-D-20-00263_Revision_1 [file giab011_giga-d-20-00263_revision_1.pdf]

GigaScience

The case for using Mapped Exonic Non-Duplicate (MEND) reads when reporting RNA sequencing depth: examples from pediatric cancer datasets

--Manuscript Draft--

Manuscript Number:	GIGA-D-20-00263R1
Full Title:	The case for using Mapped Exonic Non-Duplicate (MEND) reads when reporting RNA sequencing depth: examples from pediatric cancer datasets
Article Type:	Technical Note

Funding Information:

American Association for Cancer Research (NextGen Grant for Transformative Cancer Research)	Dr Olena M Vaske
St. Baldrick's Foundation (Consortium Award and Emily Beazley Kures for Kids Fund Hero Award)	Dr David Haussler
Alex's Lemonade Stand Foundation for Childhood Cancer (US)	Dr David Haussler
Unravel Pediatric Cancer	Dr David Haussler
Team G Childhood Cancer Foundation	Dr David Haussler
California Initiative to Advance Precision Medicine	Dr David Haussler
Live for Others Foundation	Dr David Haussler
The Schmidt Futures Foundation	Dr David Haussler
Howard Hughes Medical Institute	Dr David Haussler
Colligan Presidential Chair in Pediatric Genomics	Dr Olena M Vaske

Abstract:

Background
The reproducibility of gene expression measured by RNA sequencing (RNA-Seq) is dependent on the sequencing depth. While unmapped or non-exonic reads do not contribute to gene expression quantification, duplicate reads contribute to the quantification but are not informative for reproducibility. We show that Mapped, Exonic, Non-duplicate (MEND) reads are a useful measure of reproducibility of RNA-Seq datasets utilized for gene expression analysis.

Findings
In bulk RNA-Seq datasets from 2179 tumors in 48 cohorts, the fraction of reads that contribute to the reproducibility of gene expression analysis varies greatly. Unmapped reads constitute 1-77% of all reads (median (med.) 3%; IQR 3%); duplicate reads constitute 3-100% of mapped reads (med. 27%; IQR 30%); and non-exonic reads constitute 4-97% of mapped, non-duplicate reads (med. 25%; IQR 21%). Mapped, Exonic, Non-duplicate (MEND) reads constitute 0-79% of total reads (med. 50%; IQR 31%).
Conclusions
Since not all reads in a RNA-Seq dataset are informative for reproducibility of gene expression measurements, and the fraction of reads that are informative varies, we propose reporting a dataset's sequencing depth in MEND reads, which definitively inform the reproducibility of gene expression, rather than total, mapped or exonic reads. We provide a Docker image containing 1) the existing required tools (RSeQC, sambamba and samblaster) and 2) a custom script. We recommend that all RNA-Seq gene expression experiments, sensitivity studies and depth recommendations use MEND units for sequencing depth.

Corresponding Author:
Holly Beale, Ph.D.
University of California, Santa Cruz
Santa Cruz, CA UNITED STATES

Corresponding Author Secondary

Powered by Editorial Manager® and ProduXion Manager® from Aries Systems Corporation

Information:	
Corresponding Author's Institution:	University of California, Santa Cruz
Corresponding Author's Secondary Institution:	
First Author:	Holly Beale, Ph.D.
First Author Secondary Information:	
Order of Authors:	Holly Beale, Ph.D.
	Jacquelyn M Roger
	Drew K. A. Thomson
	Katrina Learned
	A. Geoffrey Lyle
	Ellen T Kephart
	Rob Currie
	Du Linh Lam
	Lauren Sanders
	Jacob Pfeil
	John Vivian
	Isabel Bjork
	Sofie Salama
	David Haussler
	Olena M Vaske
Order of Authors Secondary Information:	
Response to Reviewers:	December 27, 2020<br><br>Scott C. Edmunds, PhD<br>Editor<br>Gigascience<br><br>Dear Dr. Edmunds and colleagues,<br><br>Thank you for the opportunity to improve and resubmit our manuscript, “The case for using Mapped, Exonic, Non-Duplicate (MEND) reads” for consideration for publication as a Gigascience Technical Note. We have retitled the paper "The case for using Mapped Exonic Non-Duplicate (MEND) reads when reporting RNA sequencing depth" in response to reviewer feedback.<br><br>Below we have detailed our responses to the recommendations by the reviewers. The largest change is the omission of the analysis of the correlation of read counts to gene counts. Reviewer two noted that using TPM thresholds confounded the results. We agreed. Our subsequent analysis showed that even using gene-level count thresholds was confounded by including duplicates in gene-level counts but not in MEND counts. Ultimately, we realized that this analysis was not central to our argument: By definition, reads that are not mapped to exons do not contribute to reproducibility; previous studies (e.g. Fu et al) have shown that duplicate reads can be artifactual; our work shows that the fraction of unmapped, duplicate and non-exonic reads varies greatly in RNA-Seq datasets, and thus only reads definitely relevant to reproducibility--Mapped, Exonic, Non-duplicate (MEND) reads--should be reported when reporting a dataset's depth of sequencing.<br><br>Thank you for reviewing our revisions.

Powered by Editorial Manager® and ProduXion Manager® from Aries Systems Corporation

With best regards,
Holly Beale, PhD, on behalf of co-authors
Lead Computational Biologist
Treehouse Childhood Cancer Initiative

Reviewer 1 Comment 1: The authors suggest that in order to assess the accuracy of an RNAseq experiment, researchers should state the MEND read count for their experiment rather than total reads. I found the article interesting and well written. Just one query: could the authors include p-values for all correlations, and check the normality of their variables to ensure that Pearson correlations is the most appropriate statistic to use.

Answer: We thank the reviewer for this insight. We found that the data is not sufficiently normal to warrant a Pearson correlation. We have adopted Spearman correlations and added p values to Figure 4 (previously Figure S1), which is discussed in the paragraph 4 of the results section .

Reviewer 2: Beale and colleagues describe the utility of quantifying mapped exonic non-duplicate (MEND) reads as a way to summarise the quality and depth of RNA-seq data. In their report they use a large number of pediatric cancer studies to demonstrate how MEND is informative for QC. I think this report has some merit but I have a number of concerns and suggestions:

Reviewer 2 Comment 1: Since most readers will only go as far as the title and abstract it is imperative these are very clear. The title suggests we should "use" MEND counts for RNA-seq. I comprehend this as a recommendation to use MEND counts for all downstream analysis which is contrary to current best practices and a number of previous reports on the topic (PMID: 27156886, PMID: 30001700).

Answer: We thank the reviewer for this suggestion. We have added clarifying language to the title (the new title is “The case for using Mapped Exonic Non-Duplicate (MEND) reads when reporting RNA sequencing depth”, abstract (conclusions section) and body (paragraphs 1 and 3 of the background, paragraphs 2 and 4 of results, paragraph 8 of the conclusion) of the document to indicate that we are discussing read counts for the purpose of reporting sequencing depth, not individual gene measurements or downstream analysis such as differential expression.

Reviewer 2 Comment 2: In the abstract authors state "we propose using only definitively informative reads, MEND reads, for the purposes of asserting the accuracy of gene expression measured in a bulk RNA-Seq experiment." However the terms "using" and "accuracy" are not defined or demonstrated in the paper.

Answer: We have replaced "accuracy" with "reproducibility" throughout the document and demonstrate what we mean by reproducibility in the first paragraph of the background section.

As described in item 1, we have added statements to the title, abstract and body of the document clarifying that we are proposing using MEND counts for reporting sequencing depth, not individual gene measurements.

Reviewer 2 Comment 3: rRNA may explain the long tail of the unmapped reads. In my experience rRNA carry-over is the main source of unmapped reads in RNA-seq. This can be quantified by mapping reads to the set of rRNA genes with BWA or similar.

Answer: We agree that rRNA reads may be the cause of the high number of unmapped reads in a few datasets. We feel however that this analysis is beyond the scope of the manuscript. We're demonstrating that read type composition varies between datasets, not trying to explain the underlying causes of that variability.

Reviewer 2 Comment 4: Panels Fig 1B and Fig 2A are not mentioned in the body text.

Answer: Fig 1B is mentioned in the data description subsection of the methods, in the last sentence before the data analysis section. We fixed the reference to Figure 1A in

Powered by Editorial Manager® and ProduXion Manager® from Aries Systems Corporation

the first paragraph of the results that was intended to refer to Figure 2A.

Reviewer 2 Comment 5: Figure 3A x-axis should be percentages not proportions.

Answer: We thank the reviewer for identifying this. We have fixed it.

Reviewer 2 Comment 6: In the work around Supplemental Figure 2, the detection threshold is set as a certain TPM number but the logic behind this is not sound. If a TPM based threshold is used, then the threshold increases with increasing sequencing depth resulting in losing genes when adding more seq coverage. Rather, setting a static threshold is more logical, for example an average of 10 reads per gene across the experiment.

Answer: We thank the reviewer for this insight. We agree. We repeated the analysis with counts and counts per kb of transcript length. We found that the changes in correlation between gene expression and read depth observed in more highly expressed genes are confounded by the fact that we include duplicate reads in gene expression quantification, as is standard in the field (PMID: 27156886, PMID: 30001700) but not in MEND counts. In hindsight, we feel that this analysis is ancillary to the main point of the manuscript, and we have omitted it. (The main narrative of the manuscript is that previous work shows that two categories of reads aren't used for gene expression quantification, and a third, duplicates, can be spurious. In the survey of RNA-Seq datasets from a variety of sources, we show that the fraction of each read type varies greatly across datasets. This sufficiently supports our argument that neither mapped nor total reads are sufficient for describing the amount of data contributing to the reproducibility of a dataset).

Reviewer 2 Comment 7: MEND reads are similar to the number of assigned reads that are reported in most RNA-seq studies, with the difference that duplicate reads are discarded. What is the added benefit of reporting MEND reads as compared to simply the assigned reads?

Answer: In some datasets, nearly all reads are duplicates. If only assigned reads were considered, the datasets would appear to be high quality. We address this issue and previous studies in the paragraph 3 of the Conclusion.

Reviewer 2 Comment 8: Many of the tools used for RNA-seq quantification such as Kallisto or Salmon are extremely fast and can be run on relatively low-powered computers. What is the relative computational cost/time of calculating MEND reads?

Answer: It's (frankly) quite slow! Our next step is to make a faster version. We've added a "Computing requirements for MEND pipeline" section to the results on page 10 to describe the speed of the process and a note in the conclusion (paragraph 6) about the benefit of developing a faster version.

Reviewer 2 Comment 9: Can you suggest a rule of thumb for the number of MEND reads or %MEND reads for excluding poor quality datasets? Does applying this threshold improve the accuracy of downstream analysis?

Answer: We have added a discussion of this question to the conclusion in paragraph 7.

Reviewer 2 Comment 10: Lastly, authors make some recommendations that RNA-seq studies should report MEND reads, which on the face of it is very sensible, but this should be based on solid data. This recommendation is based largely on the observed correlation data shown in Fig 3C, but this is insufficient. Authors should consider one or more of the following to further support their recommendations:

a. Threshold analysis. Does applying MEND thresholds (omitting samples that do not meet acceptable MEND
thresholds) improve the accuracy of downstream analysis (i.e. the discovery of truly differentially expressed genes)?

b. Saturation analysis. Does the number of DEGs discovered for high %MEND datasets increase faster than low %MEND datasets as a function of increasing

Powered by Editorial Manager® and ProduXion Manager® from Aries Systems Corporation

	sequencing depth?<br><br>c. After randomly subsampling to a set read number, do low %MEND datasets exhibit poor kmer complexity? This could be quantified with kPAL (PMID: 25514851) or another kmer tool.<br><br>d. An analysis of RNA-seq with spike-ins and unique molecular identifiers (UMIs) to confirm that MEND reads are informative and reliable while duplicate reads are a source of bias. This could be done with a parallel analysis that does/doesn't include duplicate removal followed by differential analysis.<br><br>Answer: We thank the reviewer for this insight. We feel however that this analysis is beyond the scope of the manuscript. We're demonstrating that read type composition varies between datasets. By the nature of gene expression quantification methods, reads that do not map to genes will not contribute to the quantification, and excessive duplicate reads do not reflect the underlying gene expression.
**Additional Information:**	
**Question**	**Response**
Are you submitting this manuscript to a special series or article collection?	No
**Experimental design and statistics**<br><br>Full details of the experimental design and statistical methods used should be given in the Methods section, as detailed in our Minimum Standards Reporting Checklist. Information essential to interpreting the data presented should be made available in the figure legends.<br><br>Have you included all the information requested in your manuscript?	Yes
**Resources**<br><br>A description of all resources used, including antibodies, cell lines, animals and software tools, with enough information to allow them to be uniquely identified, should be included in the Methods section. Authors are strongly encouraged to cite Research Resource Identifiers (RRIDs) for antibodies, model organisms and tools, where possible.<br><br>Have you included the information requested as detailed in our Minimum Standards Reporting Checklist?	Yes

**Availability of data and materials**<br><br>All datasets and code on which the conclusions of the paper rely must be either included in your submission or deposited in publicly available repositories (where available and ethically appropriate), referencing such data using a unique identifier in the references and in the "Availability of Data and Materials" section of your manuscript.<br><br>Have you have met the above requirement as detailed in our Minimum Standards Reporting Checklist?	Yes

Powered by Editorial Manager® and ProduXion Manager® from Aries Systems Corporation

Manuscript

Click here to access/download;Manuscript;reporting RNA sequencing depth in MEND reads.pdf

The case for using Mapped Exonic Non-Duplicate (MEND) reads when reporting RNA sequencing depth: examples from pediatric cancer datasets

Authors

Holly C. Beale*, UC Santa Cruz Molecular, Cell and Developmental Biology; UC Santa Cruz Genomics Institute, hcbeale@ucsc.edu

Jacquelyn M. Roger, UC Santa Cruz School of Engineering, jackieroger96@gmail.com

Matthew A. Cattle, UC Santa Cruz School of Engineering, mcattle@ucsc.edu

Liam T. McKay, UC Santa Cruz School of Engineering, ltmckay@ucsc.edu

Drew K. A. Thomson, UC Santa Cruz School of Engineering, drkthomp@ucsc.edu

Katrina Learned, UC Santa Cruz Genomics Institute, klearned@ucsc.edu

A. Geoffrey Lyle, UC Santa Cruz Molecular, Cell and Developmental Biology; UC Santa Cruz Genomics Institute, aglyle@ucsc.edu

Ellen T. Kephart, UC Santa Cruz Genomics Institute, ekephart@ucsc.edu

Rob Currie, UC Santa Cruz Genomics Institute, rcurrie@ucsc.edu

Du Linh Lam, UC Santa Cruz Genomics Institute, linhvoyo@gmail.com

Lauren Sanders, UC Santa Cruz Molecular, Cell and Developmental Biology; UC Santa Cruz Genomics Institute, lmsh@ucsc.edu

Jacob Pfeil, UC Santa Cruz Genomics Institute, jpfeil@ucsc.edu

John Vivian, UC Santa Cruz Genomics Institute, jtvivian@gmail.com

Isabel Bjork[†], UC Santa Cruz Genomics Institute, ibjork@ucsc.edu

Sofie R. Salama[†], Dept. of Biomolecular Engineering, UC Santa Cruz Genomics Institute, Howard Hughes Medical Institute, ssalama@ucsc.edu

David Haussler[†], Dept. of Biomolecular Engineering, UC Santa Cruz Genomics Institute, Howard Hughes Medical Institute, haussler@ucsc.edu

Olena M. Vaske[†], UC Santa Cruz Molecular, Cell and Developmental Biology; UC Santa Cruz Genomics Institute, olena@ucsc.edu

* corresponding author

[†] contributed equally

Abstract

Background

The reproducibility of gene expression measured by RNA sequencing (RNA-Seq) is dependent on the sequencing depth. While unmapped or non-exonic reads do not contribute to gene expression quantification, duplicate reads contribute to the quantification but are not informative for reproducibility. We show that Mapped, Exonic, Non-duplicate (MEND) reads are a useful measure of reproducibility of RNA-Seq datasets utilized for gene expression analysis.

Findings

In bulk RNA-Seq datasets from 2179 tumors in 48 cohorts, the fraction of reads that contribute to the reproducibility of gene expression analysis varies greatly. Unmapped reads constitute

1-77% of all reads (median (med.) 3%; IQR 3%); duplicate reads constitute 3-100% of mapped reads (med. 27%; IQR 30%); and non-exonic reads constitute 4-97% of mapped, non-duplicate reads (med. 25%; IQR 21%). Mapped, Exonic, Non-duplicate (MEND) reads constitute 0-79% of total reads (med. 50%; IQR 31%).

Conclusions

Since not all reads in a RNA-Seq dataset are informative for reproducibility of gene expression measurements, and the fraction of reads that are informative varies, we propose reporting a dataset's sequencing depth in MEND reads, which definitively inform the reproducibility of gene expression, rather than total, mapped or exonic reads. We provide a Docker image containing 1) the existing required tools (RSeQC, sambamba and samblaster) and 2) a custom script. We recommend that all RNA-Seq gene expression experiments, sensitivity studies and depth recommendations use MEND units for sequencing depth.

Keywords

RNA-Seq "sequencing depth" duplicate unmapped exonic quality

Background

Assessing the reproducibility of RNA-Seq gene expression measurements has been a priority since the development of the assay [1,2]. The amount of sequencing generated from all regions

of the genome for a dataset generated from one biological sample is called the depth of sequence for that dataset. Seminal studies showed the following three effects of increasing the depth of sequencing: the convergence of measurements of the expression of individual genes in a single dataset to a consistent value [2], the increase in the number of true positive differentially expressed genes in cross-dataset comparisons [1], and an increase in correlations across platforms of fold-change measurements in cross-dataset comparisons [1]. These show how reproducibility within a dataset, between datasets, and across platforms all depend on the depth of sequence. The degree of reproducibility required depends on the experimental design; finding large fold changes across genes requires less reproducibility in gene expression values than finding smaller differences between isoforms. For comparing gene expression measurements between datasets, ENCODE recommends a minimum of 30 million mapped reads [3]; the GEUVADIS consortium study had a minimum goal of 20 million reads [4].

However, RNA-Seq data is not homogenous. Of the tens of millions of sequences (reads) in a typical RNA-Seq dataset, some reads cannot be mapped back to the reference transcriptome. Others map to genome regions outside of exons or have been duplicated by PCR during the library construction process or sequencing. Nearly all methods for quantifying gene expression in bulk RNA-Seq data count reads that align to exons in a gene; thus, unmapped and non-exonic reads do not contribute to measurements and are therefore uninformative [5,6]. Therefore, if the fraction of uninformative reads varies between datasets, using the total number of reads as a proxy for RNA-Seq gene expression reproducibility can result in inflated reproducibility estimates.

Duplicate reads may be due to either highly abundant transcripts or technical artifacts. The process of preparing RNA-Seq libraries involves PCR amplification. This step can generate duplicated identical or nearly identical reads, especially if the input amount is low. While the original read represents gene expression in the experimental system, the artifactual duplicate reads do not. However, duplicate reads are also generated by very highly expressed genes since each gene has a finite number of unique read sequences that can be generated from it [7]. Previous studies have shown that many duplicates in high quality datasets reflect gene expression, and there is strong evidence that duplicates should not be removed for the purpose of measuring the expression of individual genes [8,9]. Below we investigate the value of excluding duplicates at the dataset level when reporting on the dataset's sequencing depth.

Here we analyze 2179 bulk, paired end, polyA-selected RNA-Seq datasets to characterize the read types present in the datasets and evaluate what fraction of each dataset is unequivocally relevant to the reproducibility of gene expression measurements.

Methods

MEND read counting method

Quantification of Mapped, Exonic, Non-Duplicate (MEND) reads was previously described [10]. Briefly, input to the program that computes MEND is a genome-aligned bam file containing RNA-Seq read data. Duplicates are marked with Samblaster v0.1.22 [11], and the RSeQC v2.7.10 [12] script read_distribution.py quantifies exonic read and tag counts, excluding QC fail and duplicate reads as well as secondary alignments. The script parseReadDist.R, which we wrote, estimates the number of MEND reads based on RSeQC output by summing the tag

counts in CDS exons, 5' UTR exons and 3' UTR exons and multiplying by reads per tag. Since a pair of reads provides information about two nearby sequences, read counts are reported in pairs. For example, 20 million reads means that there are 20 million pairs of reads. The process for estimating MEND read counts is available as a stand-alone docker image [13] and can be executed on CodeOcean [14]. The source code is freely available on GitHub [15].

Data description

Here we discuss 2179 publicly available, polyA-selected, bulk RNA-Seq datasets we gathered for the RNA-Seq compendium [16] used for comparative single-patient analysis [10]. Accession numbers, clinical metadata and read counts for each dataset are in Table S1. Repositories and cohort information is aggregated in Tables S2 and S3.

Of the 2179 datasets, 2018 were generated from pediatric/adolescent/young adult cancer tumors, 66 were from adult cancer tumors, and 95 were from cancer tumors of individuals with unknown ages, where adults are defined as being over 30 years of age. Of the 1692 datasets with reported gender of the patient, 42% were female and 58% were male. Of the 602 datasets with reported race of the patient, 27 patients were Asian, 70 were Black/African American, 3 were Native Hawaiian or Other Pacific Islander, 494 were White and 7 were Other without further definition. None were American Indian or Alaskan Native. Of 861 datasets with reported results of the patient's Hispanic or Latino identity, 128 were Hispanic or Latino. The source tumors represent a variety of hematologic and solid malignancies (Table 1).

Table 1: Diseases represented in studied datasets

Disease	n	percent

Acute lymphoblastic leukemia	680	31.2%
Acute myeloid leukemia	221	10.1%
Medulloblastoma	201	9.2%
Glioma	193	8.9%
Osteosarcoma	157	7.2%
Acute megakaryoblastic leukemia	103	4.7%
Ependymoma	98	4.5%
Ewing sarcoma	70	3.2%
Rhabdoid tumor	65	3.0%
Rhabdomyosarcoma	53	2.4%
Lymphoma	49	2.2%
Embryonal rhabdomyosarcoma	42	1.9%
Alveolar rhabdomyosarcoma	40	1.8%
Glioblastoma multiforme	29	1.3%
Choroid plexus carcinoma	25	1.1%
Synovial sarcoma	22	1.0%
Other	131	6.0%

The datasets came from five repositories (Table S2). Each was assigned to a cohort based on 1) project accession (for EGA and SRA datasets), 2) disease sub-study for NCI Therapeutically Applicable Research to Generate Effective Treatments (TARGET), or 3) disease for datasets in the St Jude Cloud. Cohorts were assigned IDs in descending order of size. Cohort assignments were intended to approximate a typical sequencing project performed by one research group at one sequencing center. The cohorts range in size from 3 to 337 datasets (Fig. 1A); the median number of datasets in a cohort is 24.5.

All libraries were prepared with polyA selection. All data were generated via paired-end Illumina sequencing technology. The median sequence length is 101 bases (Fig. 1B).

Data analysis

RNA-Seq read data was aligned to the genome with the TOIL RNA-Seq pipeline previously described [17]. Briefly, adapters were removed with CutAdapt v1.9. Reads were then aligned with STAR v2.4.2a with indices based on GRCh38 and gencode v23. RSEM v1.2.25 was used to quantify gene expression. The source code of the pipeline is available [18]. MEND read counts were calculated with MEND qc release v1.1.1.

Read count and gene expression analysis was conducted with the R programming language, using the following packages: tidyverse, janitor, knitr, corr, cowplot, RColorBrewer, pander, kableExtra, and snakecase [19–28]. The source code used to generate the figures and statements in this manuscript is available on Github [29] and can be run and modified on CodeOcean [30].

Results

Read types in RNA-Seq data

We interrogated the read types present in our RNA-Seq datasets as defined by our gene expression quantification pipeline (Fig. 2A). We obtained the number of total and mapped reads from the aligner log. We marked duplicates in the aligned BAM file, and counted them, along with exonic reads, using RSeQC. Duplicate reads are reported as a fraction of mapped reads, and exonic reads are reported as a fraction of non-duplicate reads. The datasets ranged in total

sequence depth from 0.2 to 668 million reads, with a median value of 61 million and an interquartile range (IQR) of 53 million.

Most RNA-Seq datasets contain a small percentage of unmapped reads (Fig. 2B). While the fraction of unmapped reads in the 2179 datasets ranges from 1-77%, the median value and IQR are both 3%. The distribution is left-skewed with a long right tail (Fig. 3A). In 77 datasets, more than 25% of reads are unmapped. The value of excluding unmapped reads from sequencing depth read counts is self-evident, as these reads do not correspond to any known expressed gene and do not contribute to gene expression measurements. Including those reads in any measure of the reproducibility of gene expression measurement would misguide the researcher.

The percentage of mapped reads that are duplicate reads ("percent duplicates") is more varied than the percentage of unmapped reads. Duplicate reads constitute 3-100% of mapped reads (median 27%; IQR 30%). 426 datasets have more than 50% duplicates (Fig. 3A). The duplicate read fraction varies within and between cohorts (Fig. 3B). For example, Cohort 4 is characterized by high duplicate fractions, with 72 of the 127 datasets having more than 98% duplicates. Remarkably, these 72 datasets all identify fewer than 100 expressed genes. However, Cohort 4 does not account for all datasets with high duplicate fractions: 20 datasets in other cohorts have more than 90% duplicates. Even cohorts with generally low duplicate fractions can contain anomalous datasets; of the 41 cohorts with a median of less than 50% duplicates, 26 contain at least one dataset with more than 50% duplicates.

If duplicate reads were only a function of datasets being especially deeply sequenced, we would expect datasets with deeper sequencing to have a greater fraction of duplicate reads than all

datasets with lower depth of sequence.The total sequencing depth has a 0.52 Spearman correlation with the fraction of duplicate reads (Fig. 4). The incomplete explanation of duplicate fractions by sequence depth is consistent with Fu et. al. [8] and with the large number of datasets in Figure 4 that have very different duplicate fractions in spite of similar total read counts. The fraction of duplicate reads cannot be inferred from the total read depth.

Like percent of duplicates, the percent of non-exonic reads among all mapped, non-duplicate reads ("percent non-exonic") has a broad distribution compared to other read type fractions, ranging from 4-97% with a median of 25% and an IQR of 21%. 330 datasets have a fraction of non-exonic reads above 50%. Therefore, percentage of duplicate reads or non-exonic reads among all mapped, non-duplicate reads, are not directly informative about the dataset's gene expression measurements.

Computing requirements for MEND pipeline

We recorded the time required to run our most recent pipelines on computers with 64GB of memory and 12 VCPU. The 382 RNA-Seq datasets examined were obtained from SRA and EGA and had reads that were 100 bases in length. For datasets within 10 million total reads of the median total read size in our survey of 2179 datasets (61 million total reads), the median duration is 290 minutes (4.8 hours) for the expression pipeline and 143 minutes (2.4 hours) for the MEND pipeline.

Conclusion

Researchers wish to know that their data is sufficient for making reproducible measurements. Here we show that, for the purpose of determining whether an RNA-Seq dataset is sufficient for reproducibly measuring expression of known genes, the fraction of relevant content of an RNA-Seq dataset (percent of MEND reads) varies substantially within and between cohorts.

This work was performed using data from pediatric tumor datasets as part of the development of our comparative RNA-Seq assay for pediatric cancer patients [10,16]. Since the factors that reduce the quality of RNA-Seq datasets (e.g. degradation, low input amounts, contamination, and low base quality) are not specific to pediatric cancer datasets, we predict that other kinds of RNA-Seq datasets would also show compositional variability. The MEND read counting tool is independent of species and genome version; it can be used on any bulk RNA-Seq dataset.

Previous studies have shown that paired end libraries contain relatively few artifactual duplicate reads [7,9]. However, these studies are typically conducted on high quality datasets from a single source. For example, Parekh et. al. [9] base their conclusions on analysis of paired end datasets with a range of duplicates of 6-19%. In our survey of real-world data, cohort 4 contains 72 datasets with more than 98% duplicates, and 20 more datasets from other cohorts contain more than 90% duplicates.

There are several reasons why a survey of this breadth has not been previously performed. Obtaining and processing clinical datasets from multiple sources is an intensive effort [31]. Access to tumor datasets is usually controlled, and obtaining the 48 cohorts we report on here

required multiple legal agreements [31]. Analyzing read types requires genome-aligned reads; the files containing genome-aligned reads are large and are not generated when using the much faster pipelines that quantify gene expression via pseudoalignment. Large RNA-Seq cohorts such as GTEX and TCGA use consistent methods and exclude datasets that fail their stringent and consistent quality control [32,33]. They lack the kind of heterogeneity observed in data cohorts gathered from diverse sources. In short, generating this data for more than 2000 datasets is time-consuming, expensive, and requires staff with diverse expertise.

Measuring the number of MEND reads in a dataset is specific to the alignment parameters and gene model. We use Gencode v23, which is inclusive, defining more than 60,000 genes. By default, the aligner we use, STAR, defines reads that map to as many as 20 positions as mappable. If we changed our pipeline, asking STAR to exclude reads mapping to more than 2 positions and using a more conservative gene model with 30,000 genes, the same dataset would have fewer MEND reads due to the loss of reads that map to too many places or map only to regions newly defined as non-exonic.

In addition to being sensitive to reference files, MEND counts are slow to compute, increasing the duration of our RNA-Seq pipeline by 50%. It would be valuable to create a faster utility that takes raw reads rather than aligned reads as input. The reference-dependence could also be addressed by including a default set of references, with support for alternate ones.

Researchers planning RNA-Seq experiments look for guidance on how much sequencing their experiment requires. For comparing gene expression measurements between datasets, ENCODE recommends a minimum of 30 million mapped reads [3]; the GEUVADIS consortium

study had a minimum goal of 20 million reads [4]. However, of the 2078 datasets in this study with more than 30 million mapped reads, 16% contain fewer than 25% informative (MEND) reads. We speculate that these guidelines were not intended to include those datasets, some of which measure fewer than 100 genes. Since the median fraction of MEND reads in our survey was 50%, we recommend that a user who, for example, wants to follow the ENCODE recommendation of the depth of 30 million mapped reads ensure that they have at least 15.5 million MEND reads (a dataset with 30 million mapped reads typically has 1 million additional unmapped reads). 13% (261) of the datasets in our study that satisfy the ENCODE guideline have fewer than 50% MEND reads; 6% (134) have fewer than 10% MEND reads.

Based on these results, we recommend that 1) publications reporting the results of an RNA-Seq study with gene expression applications should report the depth of sequence as the number of MEND reads present in each dataset; 2) sensitivity studies should include read type fractions and report on the relationship between MEND reads and the measured outcome; and 3) sequencing depth recommendations should be based on MEND reads rather than total or total mapped reads.

Availability of supporting source code and requirements

Project name: MEND QC

Project home page: https://github.com/UCSC-Treehouse/mend_qc

Operating system(s): Platform independent

Programming language: Bash and R

Other requirements: Docker

License: MIT

Availability of supporting data

Accession numbers, clinical data and read counts for 2179 publicly available, bulk RNA-Seq datasets are in Supplementary Table S1. The sequence data is controlled access and can be requested via the accession numbers at the repositories in Supplementary Table S2.

Acknowledgements

We acknowledge the work of all our colleagues at the UC Santa Cruz Genomics Institute; the Computational Genomics Lab has provided an invaluable base for this work, allowing us to analyze large data sets relevant to pediatric cancer research. We thank Alejandro Sweet-Cordero and Alex G. Lee for valuable feedback on MEND analysis. We thank the many researchers who shared their sequence data [34]. Finally, we honor and thank all the children and adults who consented to donate their data to advance cancer research.

Ethics

The UCSC Institutional Review Board (IRB) has determined that our use of previously released sequence data does not constitute human subject research, and therefore does not require an IRB review.

Competing Interests

The authors declare no potential conflicts of interest.

Author Contributions

Analysis and manuscript authorship: HCB, JMR, MAC, LTM, DKAT

MEND pipeline development and integration and manuscript review: RC, DLL, JV

Data access, data processing and manuscript review: KL, ETK, LS, JP, AGL and IB

Scientific oversight and manuscript review: DH, SRS and OMV

Funding

This study was funded by American Association for Cancer Research NextGen Grant for Transformative Cancer Research Award (OMV), St Baldrick's Foundation Consortium Award and Emily Beazley Kures for Kids Fund Hero Award, Alex's Lemonade Stand Foundation for Childhood Cancer Research, Unravel Pediatric Cancer, Team G Childhood Cancer Foundation, California Initiative to Advance Precision Medicine, Live for Others Foundation, The Schmidt Futures Foundation (DH). DH is a Howard Hughes Medical Institute Investigator. OMV holds a Colligan Presidential Chair in Pediatric Genomics.

Figure titles and legends

Figure 1: RNA-Seq datasets from 48 tumor cohorts with a variety of read lengths were analyzed. A. Distribution of number of datasets per cohort. B. Distribution of length of paired-end reads in this study.

Figure 2: RNA-Seq datasets include 4 main types of sequencing reads. A. Simplified schematic illustrating read types. The X axis (blue) is a genomic locus containing an exon. The other boxes

each represent one sequencing read. Two of five reads are MEND reads. Other reads do not map to the genome (Unmapped; orange border), map to a non-exonic region of the genome (Non-exonic; green border), or are duplicates of other reads (Duplicate; red border). The MEND reads (black) fit none of these categories and are most informative for determining the reproducibility of gene expression quantification. B. Schematic illustrating read type quantification. Bars representing uninformative reads are white with a colored border. For each informative fraction, the range and median (med.) are reported.

Figure 3: Read type fractions vary within and between cohorts. A. The percent distribution of different uninformative read types observed in 2179 datasets. B. The percentage of read types observed in cohorts, annotated with the number of datasets in the cohort.

Figure 4. Duplicate fraction is not completely explained by total read depth. The Spearman correlation and p value are shown. Many datasets have very different duplicate fractions in spite of similar total read counts; n=2179..

Abbreviations

IQR: interquartile range; med.: median; MEND: Mapped, Exonic, Non-Duplicate; RNA-Seq: RNA-Sequencing.

Additional files

Table S1. Accession numbers, clinical data and read counts for 2179 publicly available, bulk RNA-Seq datasets. The accession numbers are the definitive sources; the DOI links to citations are provided for convenience.

Table S2. Sequence data repositories, URLs and abbreviations.

Table S3. Cohort names, code, repositories and dataset counts.

Bibliography

1. Marioni JC, Mason CE, Mane SM, Stephens M, Gilad Y. RNA-seq: an assessment of technical reproducibility and comparison with gene expression arrays. *Genome Res*. 2008; doi: 10.1101/gr.079558.108.
2. Mortazavi A, Williams BA, McCue K, Schaeffer L, Wold B. Mapping and quantifying mammalian transcriptomes by RNA-Seq. *Nat Methods*. 2008; doi: 10.1038/nmeth.1226.
3. ENCODE Project Consortium. Encode Standards, Guidelines and Best Practices for RNA-Seq. 2011;
4. ’t Hoen PAC, Friedländer MR, Almlöf J, Sammeth M, Pulyakhina I, Anvar SY, et al.. Reproducibility of high-throughput mRNA and small RNA sequencing across laboratories. *Nat Biotechnol*. 2013; doi: 10.1038/nbt.2702.
5. Bray NL, Pimentel H, Melsted P, Pachter L. Near-optimal probabilistic RNA-seq quantification. *Nat Biotechnol*. 2016; doi: 10.1038/nbt.3519.
6. Li B, Dewey CN. RSEM: accurate transcript quantification from RNA-Seq data with or without a reference genome. *BMC Bioinformatics*. 2011; doi: 10.1186/1471-2105-12-323.
7. Klepikova AV, Kasianov AS, Chesnokov MS, Lazarevich NL, Penin AA, Logacheva M. Effect of method of deduplication on estimation of differential gene expression using RNA-seq. *PeerJ*. 2017; doi: 10.7717/peerj.3091.
8. Fu Y, Wu P-H, Beane T, Zamore PD, Weng Z. Elimination of PCR duplicates in RNA-seq and small RNA-seq using unique molecular identifiers. *BMC Genomics*. 2018; doi: 10.1186/s12864-018-4933-1.
9. Parekh S, Ziegenhain C, Vieth B, Enard W, Hellmann I. The impact of amplification on differential expression analyses by RNA-seq. *Sci Rep*. 2016; doi: 10.1038/srep25533.
10. Vaske OM, Bjork I, Salama SR, Beale H, Shah AT, Sanders L, et al.. Comparative Tumor RNA Sequencing Analysis for Difficult-to-Treat Pediatric and Young Adult Patients With Cancer. *JAMA Netw Open*. 2019; doi: 10.1001/jamanetworkopen.2019.13968.
11. Faust GG, Hall IM. SAMBLASTER: fast duplicate marking and structural variant read extraction. *Bioinformatics*. 2014; doi: 10.1093/bioinformatics/btu314.

12. Wang L, Wang S, Li W. RSeQC: quality control of RNA-seq experiments. *Bioinformatics*. 28:2184–52012;
13. Treehouse childhood cancer initiative: ucsctreehouse/bam-mend-qc - Docker Hub. https://hub.docker.com/r/ucsctreehouse/bam-mend-qc/ Accessed 2020 Aug 21.
14. Treehouse childhood cancer initiative: CodeOcean capsule: Count Mapped, Exonic, Non-duplicate (MEND) reads in RNA-Seq data. doi.org/10.24433/CO.3151742.v1 Accessed 2020 Aug 21.
15. Treehouse childhood cancer initiative: mend_qc. https://github.com/UCSC-Treehouse/mend_qc Accessed 2020 Aug 21.
16. Treehouse childhood cancer initiative: Treehouse Public Data. https://treehousegenomics.soe.ucsc.edu/public-data/ Accessed 2020 Aug 10.
17. Vivian J, Rao AA, Nothaft FA, Ketchum C, Armstrong J, Novak A, et al.. Toil enables reproducible, open source, big biomedical data analyses. *Nat Biotechnol*. 35:3142017;
18. Treehouse childhood cancer initiative: pipelines. https://github.com/UCSC-Treehouse/pipelines Accessed 2020 Aug 21.
19. Daróczi G, Tsegelskyi R. pander: An R “Pandoc” Writer.
20. Firke S. janitor: Simple Tools for Examining and Cleaning Dirty Data.
21. Grosser M. snakecase: Convert Strings into any Case.
22. Neuwirth E. RColorBrewer: ColorBrewer Palettes.
23. R Core Team. R: A Language and Environment for Statistical Computing. Vienna, Austria: R Foundation for Statistical Computing;
24. Ruiz E, Jackson S, Cimentada J. corrr: Correlations in R.
25. Wickham H. tidyverse: Easily Install and Load the “Tidyverse.”
26. Wilke CO. cowplot: Streamlined Plot Theme and Plot Annotations for “ggplot2.”
27. Xie Y. knitr: A General-Purpose Package for Dynamic Report Generation in R.
28. Zhu H. kableExtra: Construct Complex Table with “kable” and Pipe Syntax.
29. Treehouse childhood cancer initiative: MEND_qc_survey. https://github.com/UCSC-Treehouse/MEND_qc_survey Accessed 2020 Aug 21.
30. Treehouse childhood cancer initiative: CodeOcean capsule: UCSC Treehouse MEND Survey. doi.org/10.24433/CO.4587123.v1 Accessed 2020 Aug 27.
31. Learned K, Durbin A, Currie R, Kephart ET, Beale HC, Sanders LM, et al.. Barriers to accessing public cancer genomic data. *Sci Data*. 2019; doi: 10.1038/s41597-019-0096-4.
32. GTEx Consortium. Genetic effects on gene expression across human tissues. *Nature*. 2017; doi: 10.1038/nature24277.
33. Hoadley KA, Yau C, Hinoue T, Wolf DM, Lazar AJ, Drill E, et al.. Cell-of-Origin Patterns Dominate the Molecular Classification of 10,000 Tumors from 33 Types of Cancer. *Cell*. 2018; doi: 10.1016/j.cell.2018.03.022.
34. Treehouse childhood cancer initiative: Treehouse Repository Data Acknowledgments. https://treehousegenomics.soe.ucsc.edu/public-data/acknowledgments.html Accessed 2020 Aug 10.

Figure 1

Click here to access/download;Figure;Figure_1.p

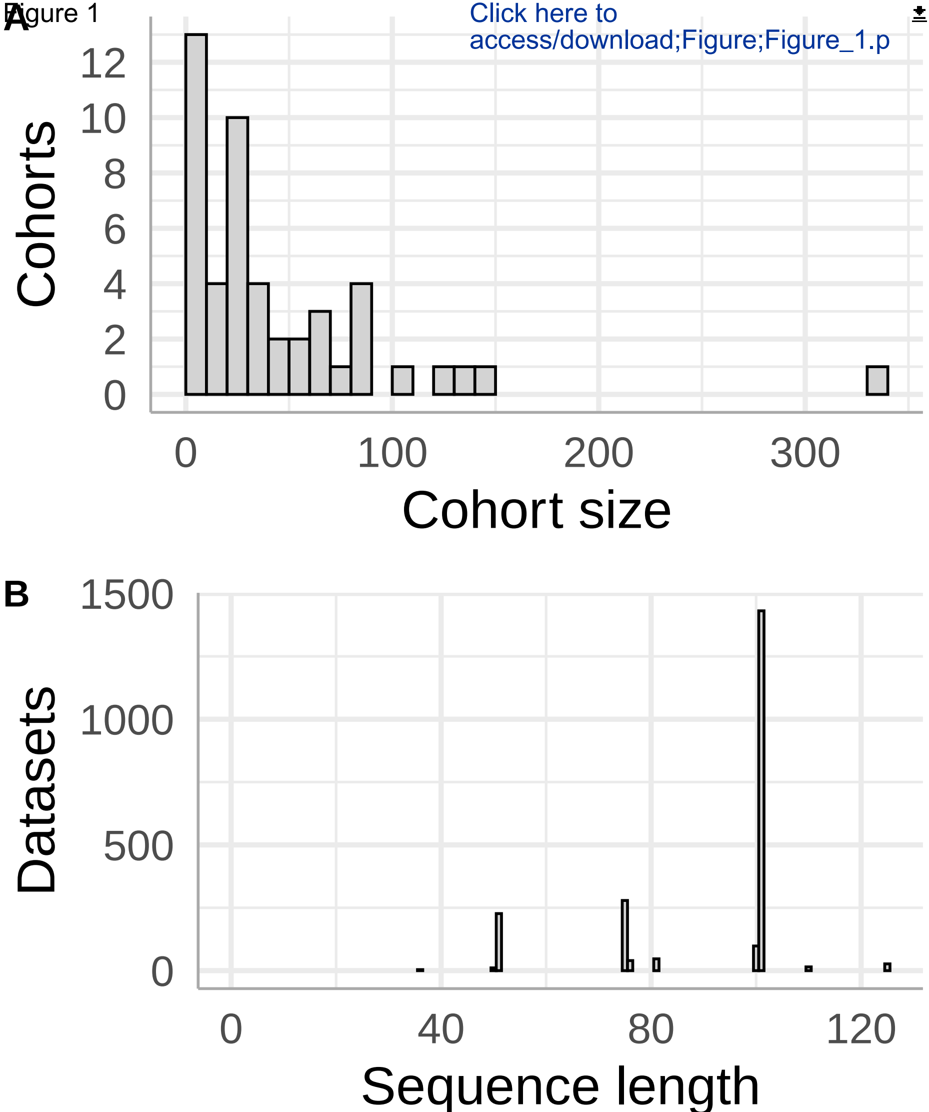

f1 2020−08−25 11:08:03

Figure 2

Click here to access/download;Figure;Figure_2.p

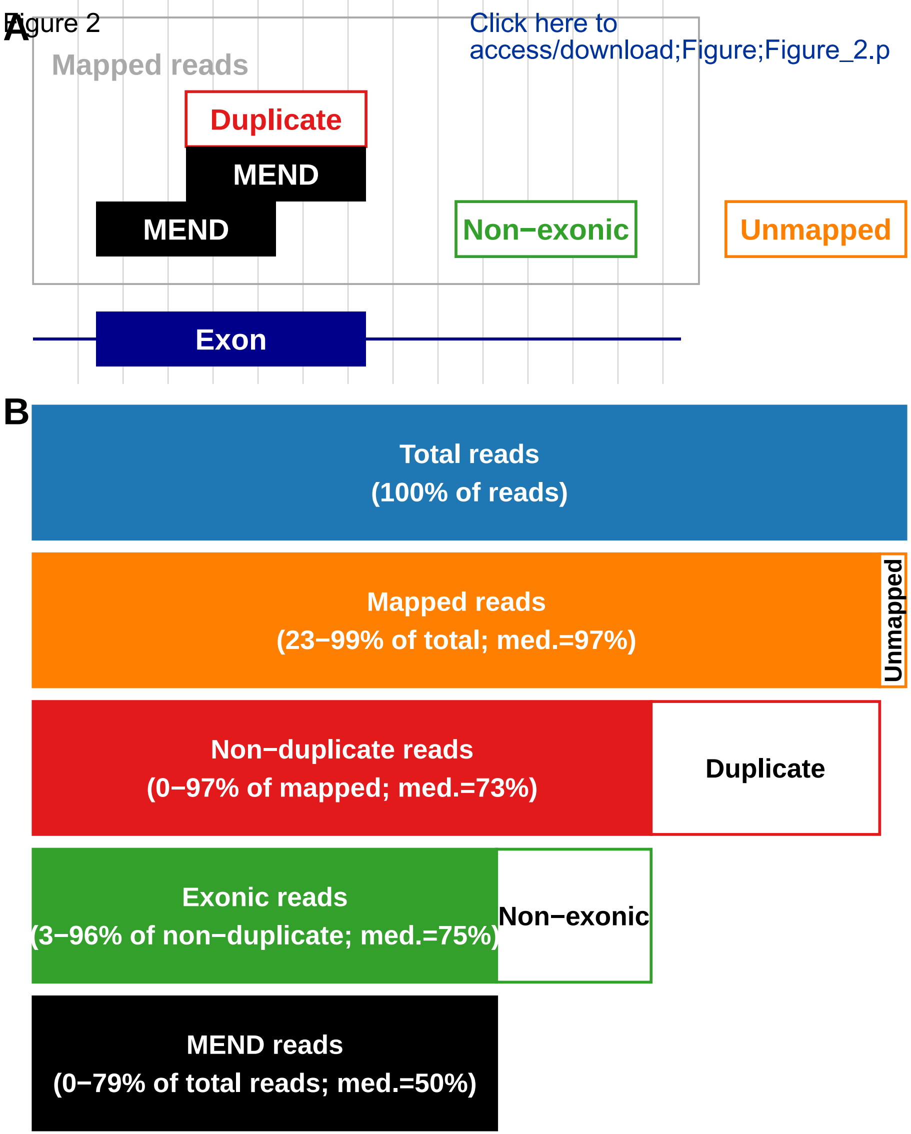

f2 2020−08−25 11:08:09

Figure 3

Click here to access/download;Figure;Figure_3.pdf

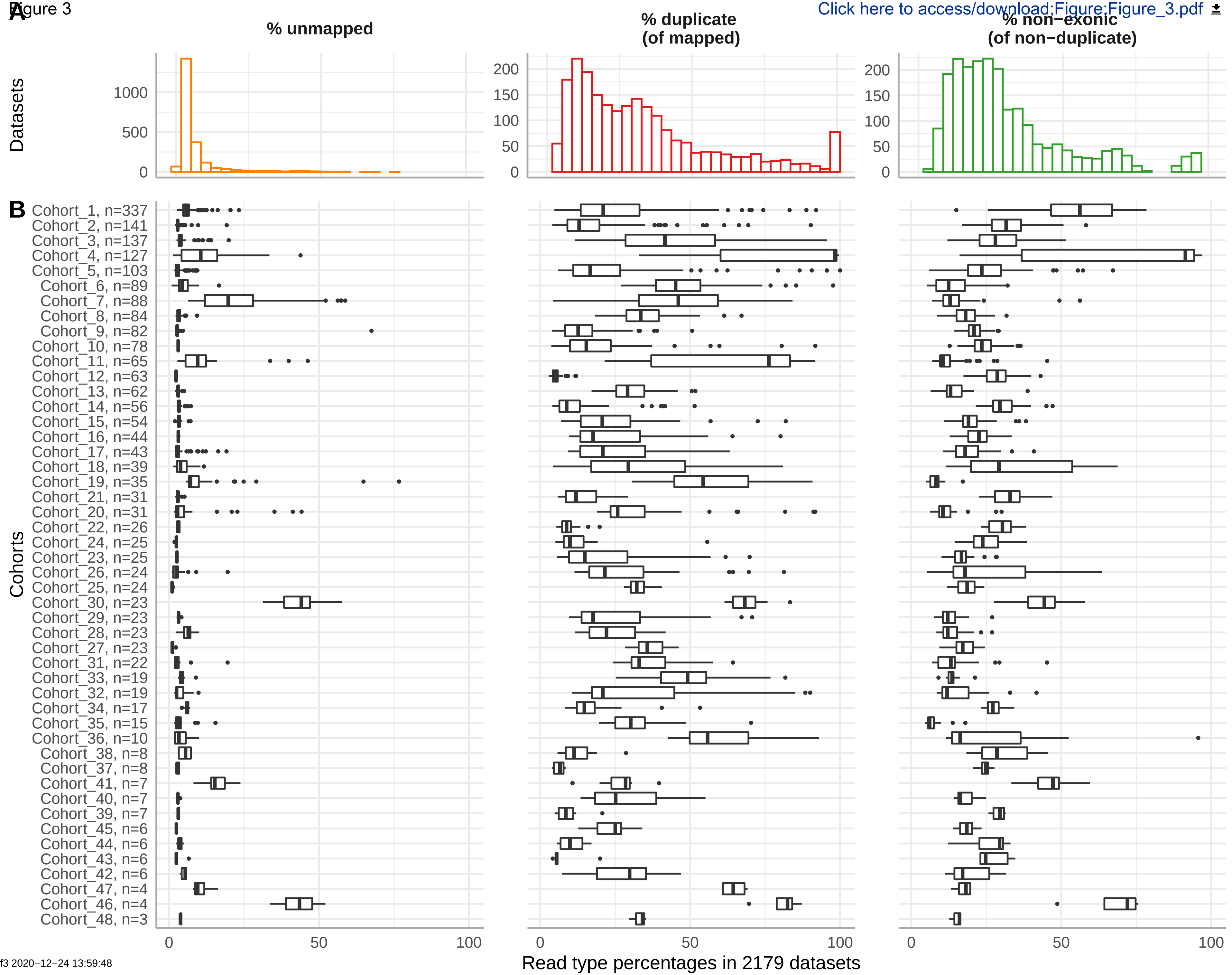

Figure 4

Click here to access/download;Figure;Figure_4.pdf

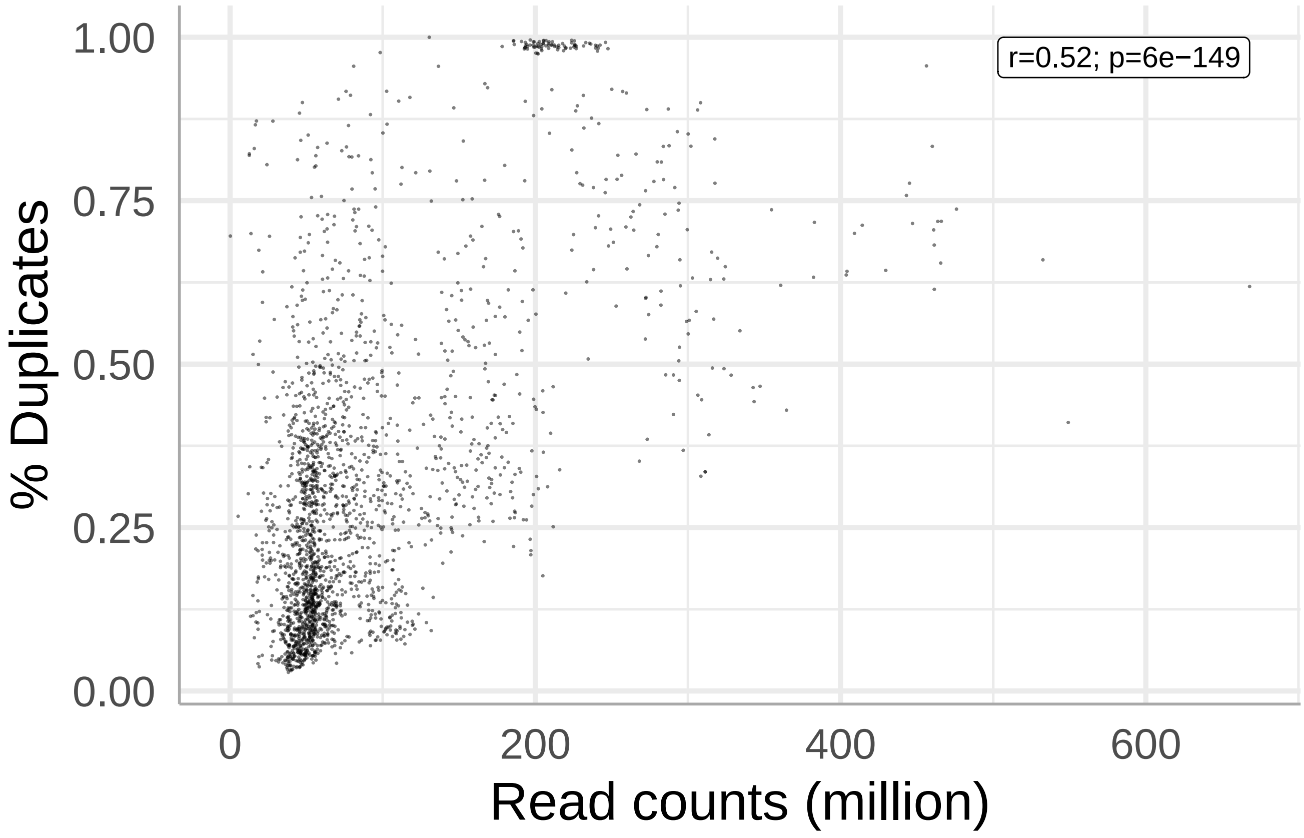

Supplementary Table S1

Click here to access/download

Supplementary Material

Table_S1.csv

Supplementary Table S2

Click here to access/download
Supplementary Material
Table_S2_repo_table.csv

Supplementary Table S3

Click here to access/download
Supplementary Material
Table_S3_cohort_table.csv
