# Supplementary material for: The case for using mapped exonic non-duplicate reads when reporting RNA-sequencing depth: examples from pediatric cancer datasets
Source: Gigascience. 2021 Mar 13;10(3):giab011. doi: 10.1093/gigascience/giab011 (PMC7955155; doi:10.1093/gigascience/giab011)
Supplement: giab011_Response_to_Reviewer_Comments_Original_Submission [file giab011_response_to_reviewer_comments_original_submission.pdf]

Author's Response To Reviewer Comments

Close

December 27, 2020

Scott C. Edmunds, PhD
Editor
Gigascience

Dear Dr. Edmunds and colleagues,

Thank you for the opportunity to improve and resubmit our manuscript, “The case for using Mapped, Exonic, Non-Duplicate (MEND) reads” for consideration for publication as a Gigascience Technical Note. We have retitled the paper "The case for using Mapped Exonic Non-Duplicate (MEND) reads when reporting RNA sequencing depth" in response to reviewer feedback.

Below we have detailed our responses to the recommendations by the reviewers. The largest change is the omission of the analysis of the correlation of read counts to gene counts. Reviewer two noted that using TPM thresholds confounded the results. We agreed. Our subsequent analysis showed that even using gene-level count thresholds was confounded by including duplicates in gene-level counts but not in MEND counts. Ultimately, we realized that this analysis was not central to our argument: By definition, reads that are not mapped to exons do not contribute to reproducibility; previous studies (e.g. Fu et al) have shown that duplicate reads can be artifactual; our work shows that the fraction of unmapped, duplicate and non-exonic reads varies greatly in RNA-Seq datasets, and thus only reads definitely relevant to reproducibility--Mapped, Exonic, Non-duplicate (MEND) reads--should be reported when reporting a dataset's depth of sequencing.

Thank you for reviewing our revisions.

With best regards,
Holly Beale, PhD, on behalf of co-authors
Lead Computational Biologist
Treehouse Childhood Cancer Initiative

Reviewer 1 Comment 1: The authors suggest that in order to assess the accuracy of an RNAseq experiment, researchers should state the MEND read count for their experiment rather than total reads. I found the article interesting and well written. Just one query: could the authors include p-values for all correlations, and check the normality of their variables to ensure that Pearson correlations is the most appropriate statistic to use.

Answer: We thank the reviewer for this insight. We found that the data is not sufficiently normal to warrant a Pearson correlation. We have adopted Spearman correlations and added p values to Figure 4 (previously Figure S1), which is discussed in the paragraph 4 of the results section .

Reviewer 2: Beale and colleagues describe the utility of quantifying mapped exonic non-duplicate (MEND) reads as a way to summarise the quality and depth of RNA-seq data. In their report they use a large number of pediatric cancer studies to demonstrate how MEND is informative for QC. I think this report has some merit but I have a number of concerns and suggestions:

Reviewer 2 Comment 1: Since most readers will only go as far as the title and abstract it is imperative these are very clear. The title suggests we should "use" MEND counts for RNA-seq. I comprehend this as a recommendation to use MEND counts for all downstream analysis which is contrary to current best practices and a number of previous reports on the topic (PMID: 27156886, PMID: 30001700).

Answer: We thank the reviewer for this suggestion. We have added clarifying language to the title (the

new title is “The case for using Mapped Exonic Non-Duplicate (MEND) reads when reporting RNA sequencing depth”, abstract (conclusions section) and body (paragraphs 1 and 3 of the background, paragraphs 2 and 4 of results, paragraph 8 of the conclusion) of the document to indicate that we are discussing read counts for the purpose of reporting sequencing depth, not individual gene measurements or downstream analysis such as differential expression.

Reviewer 2 Comment 2: In the abstract authors state "we propose using only definitively informative reads, MEND reads, for the purposes of asserting the accuracy of gene expression measured in a bulk RNA-Seq experiment." However the terms "using" and "accuracy" are not defined or demonstrated in the paper.

Answer: We have replaced "accuracy" with "reproducibility" throughout the document and demonstrate what we mean by reproducibility in the first paragraph of the background section.

As described in item 1, we have added statements to the title, abstract and body of the document clarifying that we are proposing using MEND counts for reporting sequencing depth, not individual gene measurements.

Reviewer 2 Comment 3: rRNA may explain the long tail of the unmapped reads. In my experience rRNA carry-over is the main source of unmapped reads in RNA-seq. This can be quantified by mapping reads to the set of rRNA genes with BWA or similar.

Answer: We agree that rRNA reads may be the cause of the high number of unmapped reads in a few datasets. We feel however that this analysis is beyond the scope of the manuscript. We're demonstrating that read type composition varies between datasets, not trying to explain the underlying causes of that variability.

Reviewer 2 Comment 4: Panels Fig 1B and Fig 2A are not mentioned in the body text.

Answer: Fig 1B is mentioned in the data description subsection of the methods, in the last sentence before the data analysis section. We fixed the reference to Figure 1A in the first paragraph of the results that was intended to refer to Figure 2A.

Reviewer 2 Comment 5: Figure 3A x-axis should be percentages not proportions.

Answer: We thank the reviewer for identifying this. We have fixed it.

Reviewer 2 Comment 6: In the work around Supplemental Figure 2, the detection threshold is set as a certain TPM number but the logic behind this is not sound. If a TPM based threshold is used, then the threshold increases with increasing sequencing depth resulting in losing genes when adding more seq coverage. Rather, setting a static threshold is more logical, for example an average of 10 reads per gene across the experiment.

Answer: We thank the reviewer for this insight. We agree. We repeated the analysis with counts and counts per kb of transcript length. We found that the changes in correlation between gene expression and read depth observed in more highly expressed genes are confounded by the fact that we include duplicate reads in gene expression quantification, as is standard in the field (PMID: 27156886, PMID: 30001700) but not in MEND counts. In hindsight, we feel that this analysis is ancillary to the main point of the manuscript, and we have omitted it. (The main narrative of the manuscript is that previous work shows that two categories of reads aren't used for gene expression quantification, and a third, duplicates, can be spurious. In the survey of RNA-Seq datasets from a variety of sources, we show that the fraction of each read type varies greatly across datasets. This sufficiently supports our argument that neither mapped nor total reads are sufficient for describing the amount of data contributing to the reproducibility of a dataset).

Reviewer 2 Comment 7: MEND reads are similar to the number of assigned reads that are reported in most RNA-seq studies, with the difference that duplicate reads are discarded. What is the added benefit of reporting MEND reads as compared to simply the assigned reads?

Answer: In some datasets, nearly all reads are duplicates. If only assigned reads were considered, the datasets would appear to be high quality. We address this issue and previous studies in the paragraph 3 of the Conclusion.

Reviewer 2 Comment 8: Many of the tools used for RNA-seq quantification such as Kallisto or Salmon are extremely fast and can be run on relatively low-powered computers. What is the relative computational cost/time of calculating MEND reads?

Answer: It's (frankly) quite slow! Our next step is to make a faster version. We've added a "Computing requirements for MEND pipeline" section to the results on page 10 to describe the speed of the process and a note in the conclusion (paragraph 6) about the benefit of developing a faster version.

Reviewer 2 Comment 9: Can you suggest a rule of thumb for the number of MEND reads or %MEND reads for excluding poor quality datasets? Does applying this threshold improve the accuracy of downstream analysis?

Answer: We have added a discussion of this question to the conclusion in paragraph 7.

Reviewer 2 Comment 10: Lastly, authors make some recommendations that RNA-seq studies should report MEND reads, which on the face of it is very sensible, but this should be based on solid data. This recommendation is based largely on the observed correlation data shown in Fig 3C, but this is insufficient. Authors should consider one or more of the following to further support their recommendations:

a. Threshold analysis. Does applying MEND thresholds (omitting samples that do not meet acceptable MEND
thresholds) improve the accuracy of downstream analysis (i.e. the discovery of truly differentially expressed genes)?

b. Saturation analysis. Does the number of DEGs discovered for high %MEND datasets increase faster than low %MEND datasets as a function of increasing sequencing depth?

c. After randomly subsampling to a set read number, do low %MEND datasets exhibit poor kmer complexity? This could be quantified with kPAL (PMID: 25514851) or another kmer tool.

d. An analysis of RNA-seq with spike-ins and unique molecular identifiers (UMIs) to confirm that MEND reads are informative and reliable while duplicate reads are a source of bias. This could be done with a parallel analysis that does/doesn't include duplicate removal followed by differential analysis.

Answer: We thank the reviewer for this insight. We feel however that this analysis is beyond the scope of the manuscript. We're demonstrating that read type composition varies between datasets. By the nature of gene expression quantification methods, reads that do not map to genes will not contribute to the quantification, and excessive duplicate reads do not reflect the underlying gene expression.

Close
