# Supplementary material for: The case for using mapped exonic non-duplicate reads when reporting RNA-sequencing depth: examples from pediatric cancer datasets
Source: Gigascience. 2021 Mar 13;10(3):giab011. doi: 10.1093/gigascience/giab011 (PMC7955155; doi:10.1093/gigascience/giab011)
Supplement: giab011_Response_to_Reviewer_Comments_Revision_1 [file giab011_response_to_reviewer_comments_revision_1.pdf]

Author's Response To Reviewer Comments

Close

Dear Scott and Chris-

Thank you for all your work on this manuscript. I've added all the changes suggested.

Notes from 2/1:
1) Can we add Olena Vaske (senior author) as co-corresponding author? I added that notation to the manuscript.
2) Can we add Matthew A. Cattle and Liam T. McKay (who are manuscript authors) to the dataset authorship list in the same places as they are on the manuscript author list? If this is possible, I'll also need to update the author list on the citation for the dataset. I'm sorry I didn't see this in my earlier review of the dataset citation.

Notes from 2/5:
I made updated the citation for GigaDB and also fix another error where I referred to Drew Thompson as Drew Thomson in the author list and GigaDB citation.

Thanks again!

Best regards,
Holly

Close
