# Supplementary material for: The case for using mapped exonic non-duplicate reads when reporting RNA-sequencing depth: examples from pediatric cancer datasets
Source: Gigascience. 2021 Mar 13;10(3):giab011. doi: 10.1093/gigascience/giab011 (PMC7955155; doi:10.1093/gigascience/giab011)
Supplement: giab011_Reviewer_2_Report_Original_Submission — Mark Ziemann, PhD -- 9/28/2020 Reviewed [file giab011_reviewer_2_report_original_submission.pdf]

Reviewer Report

Title: The case for using Mapped Exonic Non-Duplicate (MEND) reads when reporting RNA sequencing depth: examples from pediatric cancer datasets

Version: Original Submission **Date:** 9/28/2020

Reviewer name: Mark Ziemann, PhD

Reviewer Comments to Author:

Beale and colleagues describe the utility of quantifying mapped exonic non-duplicate (MEND) reads as a way to summarise the quality and depth of RNA-seq data. In their report they use a large number of pediatric cancer studies to demonstrate how MEND is informative for QC. I think this report has some merit but I have a number of concerns and suggestions:

1. Since most readers will only go as far as the title and abstract it is imperative these are very clear. The title suggests we should "use" MEND counts for RNA-seq. I comprehend this as a recommendation to use MEND counts for all downstream analysis which is contrary to current best practices and a number of previous reports on the topic (PMID: 27156886, PMID: 30001700).
2. In the abstract authors state "we propose using only definitively informative reads, MEND reads, for the purposes of asserting the accuracy of gene expression measured in a bulk RNA-Seq experiment." However the terms "using" and "accuracy" are not defined or demonstrated in the paper.
3. rRNA may explain the long tail of the unmapped reads. In my experience rRNA carry-over is the main source of unmapped reads in RNA-seq. This can be quantified by mapping reads to the set of rRNA genes with BWA or similar.
4. Panels Fig 1B and Fig 2A are not mentioned in the body text.
5. Figure 3A x-axis should be percentages not proportions.
6. In the work around Supplemental Figure 2, the detection threshold is set as a certain TPM number but the logic behind this is not sound. If a TPM based threshold is used, then the threshold increases with increasing sequencing depth resulting in losing genes when adding more seq coverage. Rather, setting a static threshold is more logical, for example an average of 10 reads per gene across the experiment.
7. MEND reads are similar to the number of assigned reads that are reported in most RNA-seq studies, with the difference that duplicate reads are discarded. What is the added benefit of reporting MEND reads as compared to simply the assigned reads?
8. Many of the tools used for RNA-seq quantification such as Kallisto or Salmon are extremely fast and can be run on relatively low-powered computers. What is the relative computational cost/time of calculating MEND reads?
9. Can you suggest a rule of thumb for the number of MEND reads or %MEND reads for excluding poor quality datasets? Does applying this threshold improve the accuracy of downstream analysis?
10. Lastly, authors make some recommendations that RNA-seq studies should report MEND reads, which on the face of it is very sensible, but this should be based on solid data. This recommendation is based largely on the observed correlation data shown in Fig 3C, but this is insufficient. Authors should consider one or more of the following to further support their recommendations:

a. Threshold analysis. Does applying MEND thresholds (omitting samples that do not meet acceptable MEND
thresholds) improve the accuracy of downstream analysis (i.e. the discovery of truly differentially expressed genes)?
b. Saturation analysis. Does the number of DEGs discovered for high %MEND datasets increase faster than low %MEND datasets as a function of increasing sequencing depth?
c. After randomly subsampling to a set read number, do low %MEND datasets exhibit poor kmer complexity? This could be quantified with kPAL (PMID: 25514851) or another kmer tool.
d. An analysis of RNA-seq with spike-ins and unique molecular identifiers (UMIs) to confirm that MEND reads are informative and reliable while duplicate reads are a source of bias. This could be done with a parallel analysis that does/doesn't include duplicate removal followed by differential analysis.

Level of Interest

Please indicate how interesting you found the manuscript: Choose an item.

Quality of Written English

Please indicate the quality of language in the manuscript: Choose an item.

Declaration of Competing Interests

Please complete a declaration of competing interests, considering the following questions:

- Have you in the past five years received reimbursements, fees, funding, or salary from an organisation that may in any way gain or lose financially from the publication of this manuscript, either now or in the future?
- Do you hold any stocks or shares in an organisation that may in any way gain or lose financially from the publication of this manuscript, either now or in the future?
- Do you hold or are you currently applying for any patents relating to the content of the manuscript?
- Have you received reimbursements, fees, funding, or salary from an organization that holds or has applied for patents relating to the content of the manuscript?
- Do you have any other financial competing interests?
- Do you have any non-financial competing interests in relation to this paper?

If you can answer no to all of the above, write 'I declare that I have no competing interests' below. If your reply is yes to any, please give details below.

I declare that I have no competing interests.

I agree to the open peer review policy of the journal. I understand that my name will be included on my report to the authors and, if the manuscript is accepted for publication, my named report including any attachments I upload will be posted on the website along with the authors' responses. I agree for my report to be made available under an Open Access Creative Commons CC-BY license (http://creativecommons.org/licenses/by/4.0/). I understand that any comments which I do not wish to be included in my named report can be included as confidential comments to the editors, which will not be published.

Choose an item.

To further support our reviewers, we have joined with Publons, where you can gain additional credit to further highlight your hard work (see: https://publons.com/journal/530/gigascience). On publication of this paper, your review will be automatically added to Publons, you can then choose whether or not to claim your Publons credit. I understand this statement.

Yes Choose an item.
